# Editorial: Translational development of tailored implants via advanced processing and surface modifications for tissue regeneration

**DOI:** 10.3389/fbioe.2025.1686388

**Published:** 2025-09-17

**Authors:** Mike Barbeck, Sebastian Stammkötter, Rumen Krastev, Frank Walther

**Affiliations:** ^1^ Clinic and Policlinic for Dermatology and Venereology, University Medical Center Rostock, Rostock, Germany; ^2^ Chair of Materials Test Engineering (WPT), TU Dortmund University, Dortmund, Germany; ^3^ Reutlingen University, Reutlingen, Germany

**Keywords:** biocompability, macrophage polarization, additive manufactuing, biomaterial development, implants

The field of implantable biomaterials is undergoing a pivotal evolution—from inert prosthetics to biofunctional constructs engineered to actively engage and direct host tissue responses. The Frontiers Research Topic, *Translational Development of Tailored Implants Based on New Processing Approaches and Surface Modifications for Tissue Regeneration*, highlights the centrality of implant geometry, manufacturing methods, and surface design in regulating biological outcomes and encourages sustainable and predictive preclinical development (Akbas et al., 2025; Stich et al., 2022).

Advances in surface engineering allow implants to do more than exist—they now influence immune behavior and tissue healing. Nanotopographical and chemical modifications of titanium, such as hydrophilic patterning or bioactive peptide coatings, have been demonstrated to shift macrophage polarization toward the regenerative M2 phenotype, thereby promoting early osteogenesis (Ramaglia et al., 2013; Zhang et al., 2021). Multifunctional coatings incorporating ions like zinc, magnesium, and copper, hydroxyapatite, and graphene significantly enhance osteogenic and antibacterial performance, positioning implants to combat infections while fostering bone growth (Chi et al., 2021).

Additive manufacturing (AM) provides unprecedented control of macro- and microscale architecture, enabling implants to mimic native bone structure and mechanical properties. Within the Frontiers Theme, studies detail how surface treatments such as sandblasting and acid etching on AM versus machined titanium surfaces influence osteoblast adhesion across storage conditions (Akbas et al., 2025), and how sophisticated modeling of AM Ti–6Al–4V alloys supports predictive design for clinical applications (Lee et al., 2025). Broader reviews emphasize pore size optimization and porous titanium functionality, including bionic design strategies for orthopedic implants (Stich et al., 2022).

Complementary to geometry and surface chemistry, immune modulation via biomaterial design is essential. Macrophage polarization dynamics—especially a controlled transition from the initial inflammatory M1 phenotype to regenerative M2—are critical for vascularization and matrix deposition, while excessive M1 activity can precipitate fibrotic encapsulation (Zhang et al., 2021). Nanotube arrays and nanoporous titanium surfaces direct these processes, influencing osteoblast differentiation and osteoclast inhibition via integrin–FAK–MAPK signaling (Stich et al., 2022).

Intriguingly, emerging evidence indicates that controlled inflammatory signaling may be beneficial. For instance, Cu^2+^-induced M1-like activation has been shown to trigger osteogenesis via BMP-Smad-RUNX2 pathways, challenging the binary view of inflammation (Zhang et al., 2021). Meanwhile, innovative approaches using click-chemistry to functionalize polymers or electrospun fibers are driving targeted immune modulation and reducing fibrous encapsulation, while preserving topographical cues and biocompatibility (Cifuentes and Borros, 2013).

Surface treatments like plasma oxidation and protein immobilization further refine host interaction. By modifying wettability and surface energy, plasma-treated implants enhance cell adhesion and reduce early inflammatory responses. Protein grafts—such as RGD peptides or fibronectin domains—improve biocompatibility and promote osteoblast attachment without compromising endothelial cell functions (Cifuentes and Borros, 2013).

Despite promising innovations, translating bench concepts into clinical success remains fraught with challenges. *In vitro* models often fail to account for patient-specific variables—such as systemic inflammation, impaired vascularity, or comorbidities—that critically influence healing. To bridge this gap, robust preclinical systems (e.g., large-animal models or *ex vivo* human tissues) and comprehensive clinical endpoints measuring tissue quality—not merely integration rates—must be prioritized (Stich et al., 2022).

The path forward demands a holistic and collaborative approach, with material scientists, immunologists, computational modelers, clinicians, and regulators co-designing implants. We must also update regulatory frameworks to recognize implants as active, regenerative agents within the body.

We stand at a threshold: equipped with tools and knowledge to create implants that actively communicate, heal, and adapt. If driven by rigorous interdisciplinary strategy and supported by flexible regulation, the research momentum catalyzed by the Frontiers initiative can deliver a new generation of genuinely regenerative implants.

The DFG research unit 5,250 on the topic “Mechanism-based characterization and modeling of permanent and bioresorbable implants with tailored functionality based on innovative *in vivo*, *in vitro* and *in silico* methods” (project no. 449916462, www.for5250.de) is a very good motivation for this special issue (Koek, 2025).
